# Heat stress and the chicken gastrointestinal microbiota: a systematic review

**DOI:** 10.1186/s40104-025-01225-6

**Published:** 2025-06-16

**Authors:** Chris Major Ncho

**Affiliations:** Department of Environmental Systems Science, Institute of Agricultural Sciences, ETH Zürich, Zurich, 8092 Switzerland

**Keywords:** Broiler, Climate change, Laying hens, Microbiome, Poultry, Welfare

## Abstract

Heat stress (HS) has become a significant challenge for poultry farming due to an increase in global temperatures. Existing literature suggests that the health effects of HS in chickens are related to its impact on the gastrointestinal tract. While there is evidence of the detrimental consequences of HS on the gut structure, little is known about the effects of HS on the microbial population inhabiting this organ. Fortunately, recent advancements in "omics" technologies have made investigating the interaction between HS and the gut microbiota possible. Therefore, a systematic review was conducted to assess the effects of HS on chicken gut microbiota. In July 2024, a comprehensive literature search was performed across scientific repositories, including Scopus, PubMed, Science Direct, and Google Scholar. Eighteen studies met the eligibility criteria for inclusion and a qualitative synthesis of their results was conducted according to the PRISMA guidelines. Current evidence indicates that HS poses a significant challenge to the gastrointestinal system of chickens, resulting in a range of physiological reactions. These changes trigger fierce competition among beneficial microbial species for limited nutrients, promote microbial shifts from obligate to facultative anaerobes, and increase the abundance of microbial species with high resistance to elevated environmental temperatures. Furthermore, the proliferation of pathogens is exacerbated. Ultimately, gut microbiota profiling highlights changes in microbial diversity, alterations in the composition of microbial populations, disruptions in specific microbial functional pathways (tricarboxylic acid cycle, amino acid metabolism, antioxidant biosynthesis, and fatty acid degradation), and the breakdown of complex networks that govern microbial interactions. Understanding the complex relationship between HS and microbial shifts within the chicken gut can provide valuable insights for the development of sustainable mitigation strategies. Further research is needed to expand the current knowledge and employ more advanced literature synthesis techniques such as meta-analyses.

**Graphical Abstract:**

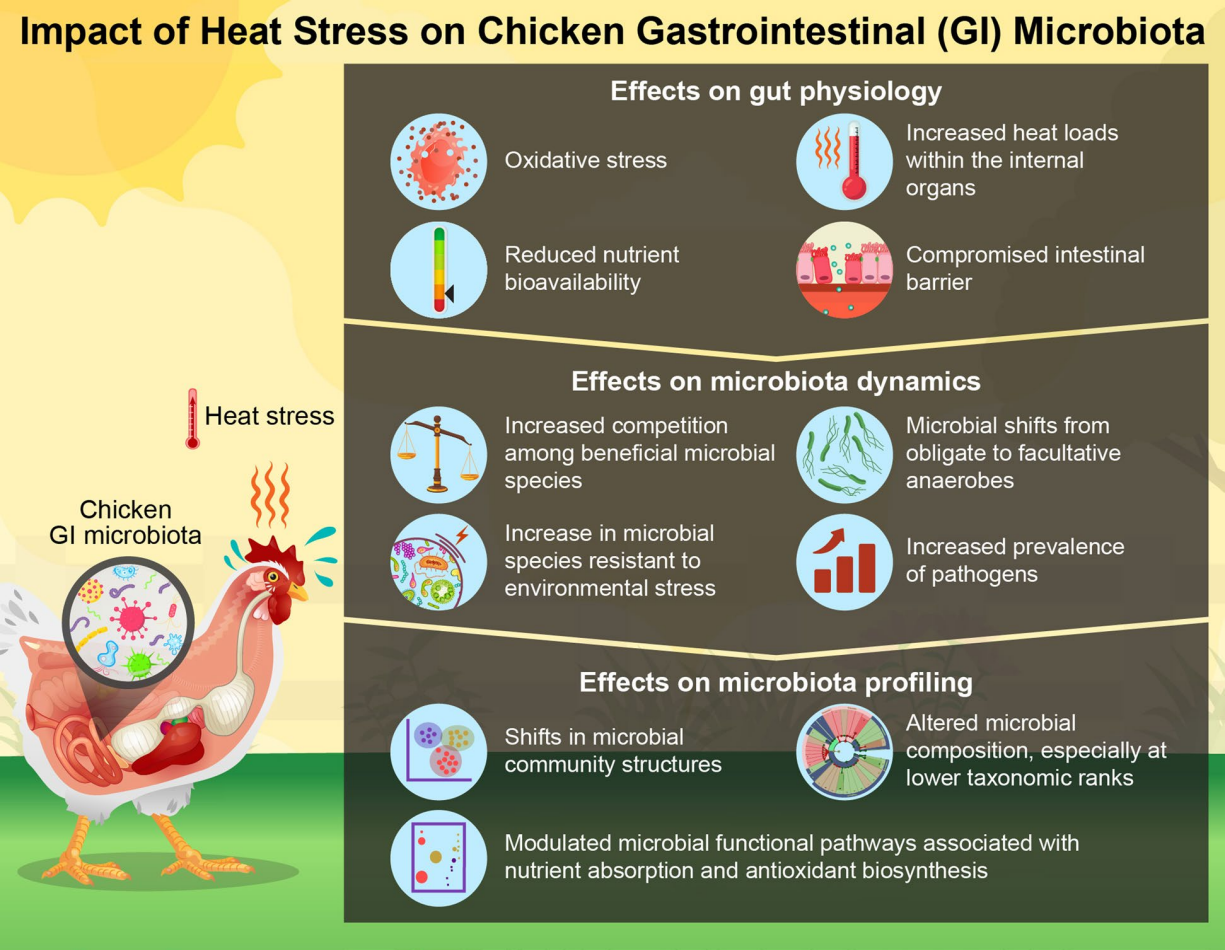

## Background

Modern chicken lines have undergone decades of growth and production [1]. Although this has resulted in optimal feed efficiency, it has also led to several drawbacks, such as low disease resistance and high sensitivity to environmental temperature variations [2]. Birds are particularly vulnerable to high ambient temperatures because of their lack of sweat glands and the presence of feathers on their bodies [3]. As a result, rising global temperatures have become a significant concern for the poultry industry because they often lead to heat stress (HS) [4]. HS is defined as the inability of an organism to maintain a balance between heat production and loss [5, 6]. To cope with the HS, birds pant and crouch while extending their wings to divert blood flow to the periphery and reduce their internal heat load [7]. However, these modifications are not always effective when HS is prolonged for extended periods [8, 9]. One of the first organs vulnerable to HS is the gastrointestinal tract [10]. Previous studies have shown that HS-induced physiological changes in poultry lead to intestinal alterations [11]. For example, several studies have reported an association between HS and the alterations in intestinal morphology, thereby resulting in the disruption of intestinal barriers [12]. These modifications result in a phenomenon known as the leaky gut syndrome, which alters the pH of the gut milieu, causes oxidative stress, and leads to inflammation [13]. Challenges to intestinal health and compromised gut integrity are also associated with severe limitations in nutrient absorption, thereby reducing overall bird performance [14].

**Fig. 1:**
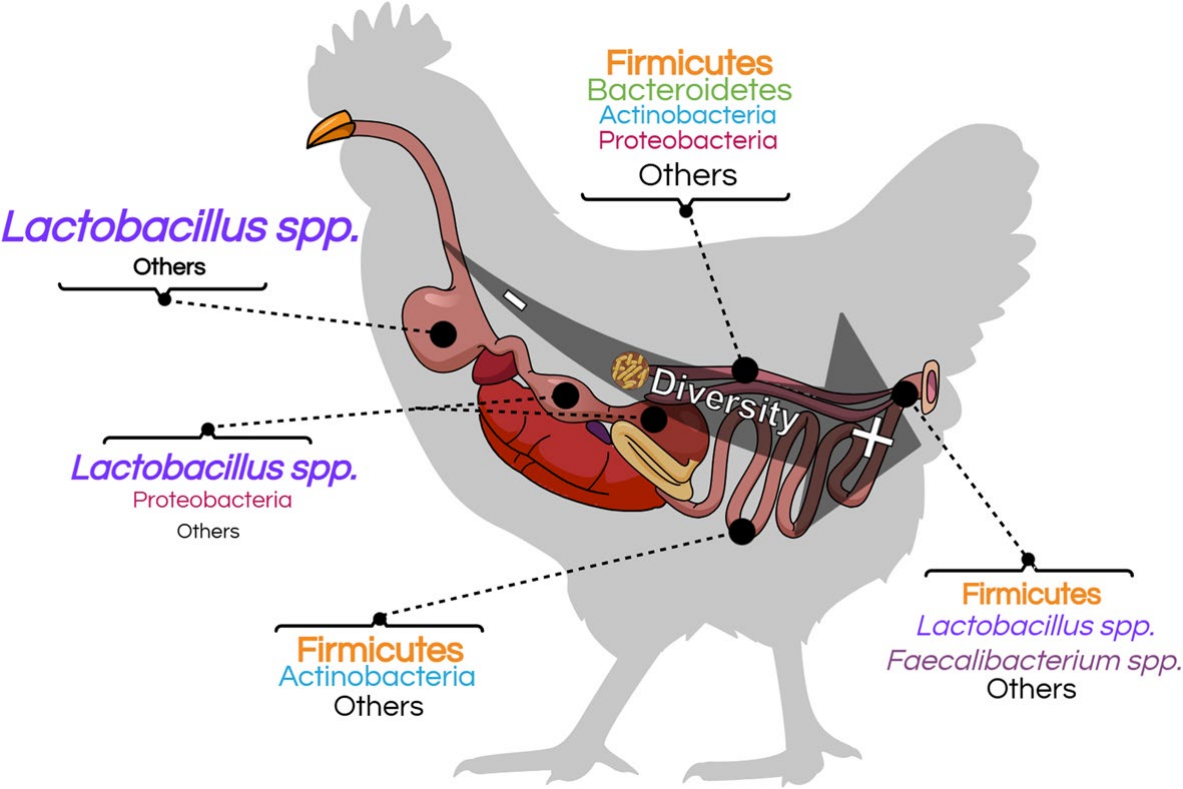
Simplified representation of the major microbial taxa inhabiting the different sections of the gastrointestinal tract of chickens. The microbiota of chicken increases in diversity from the proximal to the distal sections. The size of the microbial taxa is associated with its relative abundance. The figure has been adapted based on previously published work [22]

Advances in “omics” technologies have provided evidence of a comprehensive microbial ecosystem in the gastrointestinal tracts of animals [15]. Approximately 10^11^ bacteria were found per gram of gut content in chickens, highlighting the close relationship between these microbial populations and their host [16]. Although there is relatively low diversity at hatching, the gut microbiota of birds rapidly diversifies within the first weeks of life, with evidence of increased complexity in the microbial section of the gut as one moves from the proximal to distal parts (Fig. 1) [17]. Recent research supports the existence of substantial evidence indicating that HS can alter the composition and diversity of the intestinal microbiota [18, 19]. It is hypothesized that the reduced bioavailability of nutrients and increased internal loads associated with HS may inhibit the growth of beneficial bacteria, thereby promoting pathogen proliferation [10]. However, the results are inconsistent with the disparities reported in various studies. For instance, compared with a thermoneutral environment, acute and chronic HS were found to facilitate the translocation of pathogens in the intestines of chickens [20]. In contrast, Xing et al. [21] observed marginal alterations in the cecal microbiota composition of chickens exposed to cyclic HS conditions. Indeed, following the 14-day exposure, no significant differences were detected in the microbial population at the phylum level. Additionally, only nine taxa at the genus level were identified as potential biomarkers, several of which were non-pathogenic. Thus, confounding factors, such as the type of HS and the parameters reported across studies, may explain the differences observed.

In summary, it is essential to condense the existing body of knowledge into straightforward concepts that can guide future investigations into the function of HS in regulating the gastrointestinal microbiota of chickens. Therefore, this study aimed to examine the relationship between HS and the diversity, composition, and functional pathways of chicken gut microbiota. To achieve this objective, we conducted a systematic and reproducible literature review, followed by a thorough synthesis and analysis of the reported results. Finally, we investigated potential explanations for the observed outcomes across studies and delineated recommended directions for future research in this field.

## Methods

The current review was conducted in accordance with the Preferred Reporting Items for Systematic Reviews and Meta-Analyses (PRISMA) statement, which was developed to enhance the reporting quality of systematic reviews with an emphasis on reproducibility. The comprehensive methodology details the query formulation, data collection, processing, and qualitative appraisal of the selected studies. As recommended, the Population, Intervention, Comparison, and Outcome (PICO) approach was used as a framework for implementing the current review. The primary research question was to elucidate the potential effects of HS on the gastrointestinal tract of chickens. Specifically, the PICO components are as follows:

- Population: Animals from the *Gallus Gallus Domesticus* species encompassing all layers and broiler lines currently employed in the poultry industry.
- Intervention: HS was applied in experimental setups, including cyclic, acute, chronic, and natural exposures to ambient temperatures above the thermoneutral zones of the birds.
- Comparison: Responses of heat-stressed birds were compared with those of their thermoneutral counterparts.
- Outcomes: Commonly assessed microbiota-related metrics and parameters such as diversity, composition, and functional pathways.

As systematic reviews are designed to address specific questions, we limited the outcomes to bacterial microbiota and did not include a synthesis or discussion of other microorganisms, such as viruses and protozoa, as there are currently insufficient experimental studies describing the results on these microorganisms. In addition, only the variables evaluated in the studies included in the review are included in the discussion.

### Search strategy

One of the fundamental steps in conducting systematic reviews is to identify suitable databases that provide a substantial number of references that may include the studies of interest. Consequently, a comprehensive search was conducted in July 2024 utilizing four general-purpose scientific repositories commonly employed in animal and veterinary science: ScienceDirect, Google Scholar, PubMed, and Scopus. Queries containing keywords relevant to our research interest were systematically employed to retrieve pertinent references, which were subsequently converted and exported into the RIS format for further processing using EndNote software v21. Specifically, the full-length queries were TITLE-ABS ((heat AND stress) AND (chicken OR hens OR broiler*) AND (microbio*)) AND (LIMIT-TO (DOCTYPE, “ar”)) for Scopus and ((heat stress[Title/Abstract]) AND (chicken[Title/Abstract] OR hens[Title/Abstract] OR broiler*[Title/Abstract])) AND (microbio*[Title/Abstract]) for PubMed. An advanced search function was utilized in Google Scholar. The criteria were limited to the title of the manuscripts that contained either keywords or combinations of keywords such as “chicken”, “layer”, “heat”, “hot”, “heat stress”, “microbiome”, “microbiota”, and “microbe”. This restriction was implemented to include a manageable number of references potentially relevant to our topic because the standard parameters of Google Scholar often yield thousands of results that cannot be effectively scrutinized. A similar approach was employed in ScienceDirect with the keywords mentioned above but with restrictions on the title, abstracts, and keywords of the manuscripts. No authors have been contacted for supplementary information or unpublished data.

### Selection criteria

In systematic reviews, the scope of a study is often defined by a set of inclusion and exclusion criteria that assist in constraining the evidence to relevant studies addressing the topic of interest. This step facilitated the clarification and determination of the manuscripts selected for qualitative synthesis of the literature. The selection criteria for the current review were as follows:

(i) Studies using chickens as experimental subjects.
(ii) Studies should exclusively assess HS in their experimental designs. Consequently, studies that tested the efficacy of HS mitigation solutions were also excluded. This criterion was considered because researchers typically assess the response variable based on the anticipated mechanism of action of their mitigation strategies. For instance, some dietary supplements may influence specific microbial taxa more than others.
(iii) The study design should incorporate at least one control treatment at a thermoneutral temperature.
(iv) Studies should include at least one parameter related to microbiome analysis (microbiota diversity, microbiota composition, and microbial functional pathway analysis).
(v) There should be a section on the materials and methods for microbial DNA extraction, amplification, and sequencing. This criterion was included as it certifies the use of sequencing techniques to obtain the results presented in this study.
(vi) Microbiota analysis should be conducted on at least one component of the gastrointestinal tract (from the crop to the colon) of birds, including feces, as it may often represent the microbial composition of the colon.

Studies that did not meet all the selection criteria were excluded.

### Data extraction and processing

Although only a qualitative assessment of the literature was conducted in this review, specific characteristics of each study were collected to provide a comprehensive description of the current evidence. Unlike meta-analyses, numerical values from the results section were not collected. Instead, only metadata relevant to the current topic and general conclusions about the outcomes were gathered. Consequently, bibliographical data (author, date of publication, country of origin), characteristics of the experimental animals (lines, production purpose, age at the beginning of the trial, organs sampled for sequencing), HS protocols (maximum temperature, daily duration of exposure, overall duration of exposure, and type of exposure), and microbiota analyses (alpha diversity, beta diversity, composition, and functional profile) were collected and are reported in Table 1. Additionally, whether the authors reported significant findings concerning the microbiota analyses performed in their trials is summarized in Table 2.

**Table 1:** Characteristics of the experimental designs of the studies included in the systematic review

Study	HS max temperature, °C	HS starting age	HS duration, d	HS Category	Breed/Line	Organ sampled
Xing et al. [21]	35	28 weeks	28	Cyclic	Hy-Line (Layers)	Caecum
Shi et al. [23]	38	14 d	28	Chronic	Yellow	Caecum
Wang et al. [24]	31	28 d	14	Chronic	Arbor Acres	Ileum
Liu et al. [25]	32	21 d	14	Cyclic	Arbor Acres	Caecum
Campos et al. [26]	36	28 d	28	Cyclic	Multiple	Caecum
Emami et al. [27]	36	28 d	14	Cyclic	Multiple	Ileum
Jin et al. [28]	32.5	8 weeks	28	Cyclic	Yellow	Ileum
Liu et al. [29]	32.5	8 weeks	28	Cyclic	Yellow	Caecum
Liu et al. [30]	32.5	8 weeks	28	Cyclic	Yellow	Jejunum
Liu et al. [31]	33	28 d	14	Cyclic	Arbor Acres	Caecum
Zhu et al. [32]	34.1	340 d	46	Cyclic	Hy-Line (Layers)	Feces
Yuan et al. [33]	33	28 d	14	Cyclic	Arbor Acres	Caecum
Seo et al. [34]	32	21 d	21	Cyclic	Ross 308	Jejunum
Wang et al. [35]	30	11 weeks	70	Cyclic	Isa Brown (Layers)	Caecum
Wang et al. [36]	31	28 d	21	Chronic	Arbor Acres	Caecum
Yang et al. [37]	36.5	1 d	42	Chronic	Yellow	Caecum
Zhang et al. [38]	36	56 d	14	Cyclic	Yellow	Caecum
Zhou et al. [39]	36	12 weeks	21	Cyclic	Hy-Line (Layers)	Caecum

*HS* Heat stress

**Table 2:** Comprehensive appraisal of the outcomes assessed in the studies included in the systematic review

Study	Outcomes assessed				Results		
	Alpha diversity	Beta diversity	Microbial composition	Functional pathway	Alpha diversity		Beta diversity^b^
					Richness^a^	Evenness^a^	
Xing et al. [21]	Reported	Reported	Reported	Not reported	Non Sig	Non Sig	Sig
Shi et al. [23]	Reported	Reported	Reported	Not reported	Non Sig	Non Sig	Sig
Wang et al. [24]	Reported	Reported	Reported	Not reported	Increased	Non Sig	Sig
Liu et al. [25]	Reported	Reported	Reported	Not reported	Non Sig	Non Sig	Sig
Campos et al. [26]	Reported	Reported	Reported	Reported	Sig	Non Sig	Sig
Emami et al. [27]	Reported	Reported	Reported	Reported	Increased	Increased	Sig
Jin et al. [28]	Reported	Reported	Reported	Not reported	Non Sig	Non Sig	Sig
Liu et al. [29]	Reported	Reported	Reported	Not reported	Non Sig	Non Sig	Sig
Liu et al. [30]	Reported	Reported	Reported	Not reported	Non Sig	Non Sig	Sig
Liu et al. [31]	Reported	Reported	Reported	Reported	Increased	Increased	Sig
Zhu et al. [32]	Not reported	Not reported	Reported	Reported	NA	NA	NA
Yuan et al. [33]	Reported	Reported	Reported	Not reported	Non Sig	Non Sig	Non Sig
Seo et al. [34]	Reported	Reported	Reported	Reported	Non Sig	Non Sig	Sig
Wang et al. [35]	Not Reported	Reported	Reported	Not reported	NA	NA	Sig
Wang et al. [36]	Not reported	Not reported	Reported	Not reported	NA	NA	NA
Yang et al. [37]	Reported	Reported	Reported	Reported	Non Sig	Non Sig	Sig
Zhang et al. [38]	Reported	Reported	Reported	Not reported	Non Sig	Decreased	Sig
Zhou et al. [39]	Reported	Reported	Reported	Not reported	Non Sig	Non Sig	Sig

*NA* Not applicable, *Non Sig* Non-significant, *Sig* Significant

^a^The results indicate that at least one of the metrics utilized exhibited a statistically significant difference between the thermoneutral and heat stress groups

^b^The results of the multivariate statistical tests (i.e., PERMANOVA, ANOSIM, or Mantel test) indicated significant differences or there was evidence of separate clustering between the thermoneutral and heat stress treatments using at least one beta diversity metric (e.g., Euclidean, Jaccard, Bray-Curtis, and UniFrac)

## Results

### Study selection workflow

**Fig. 2:**
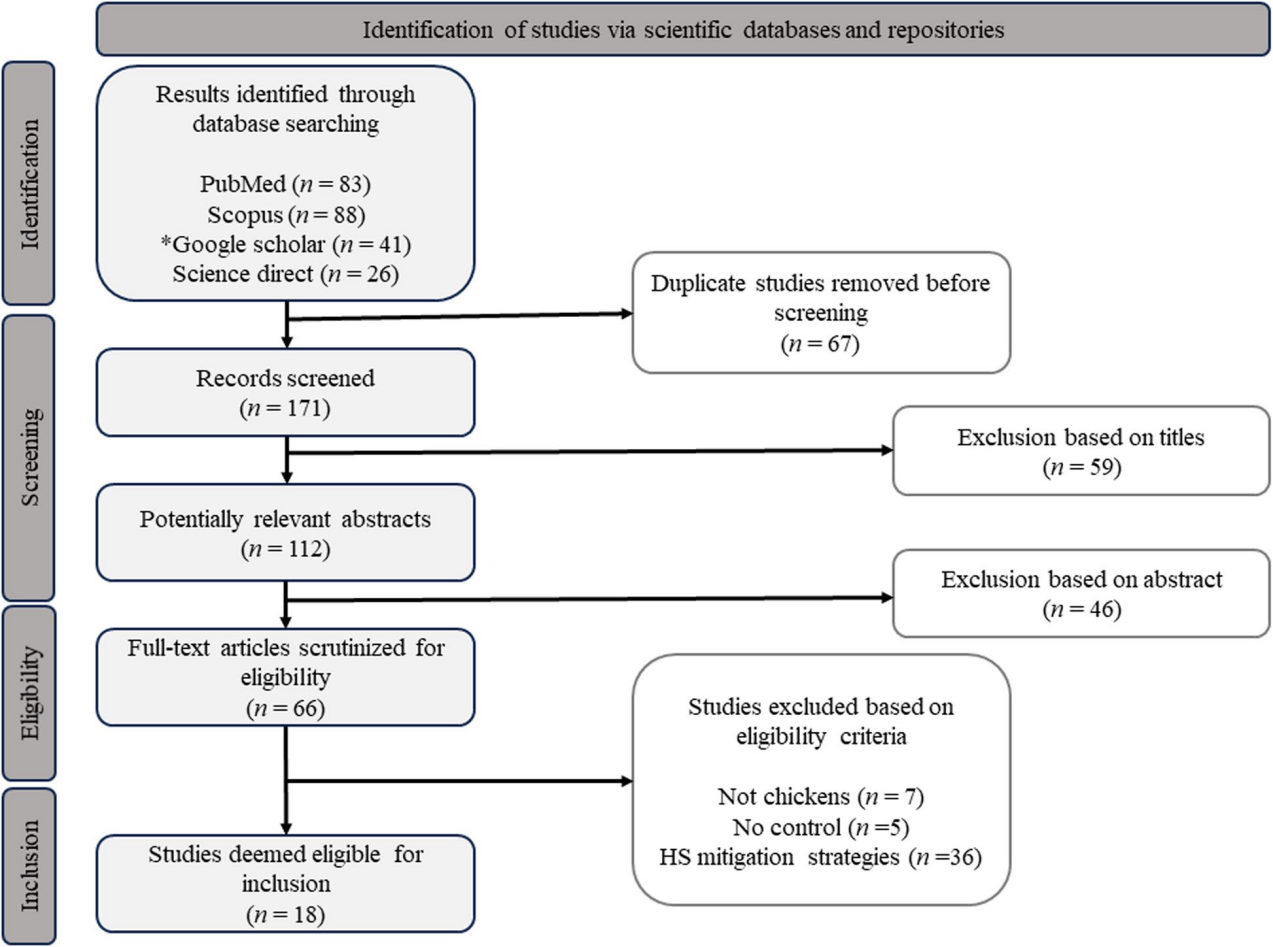
Preferred reporting items for systematic reviews and meta-analyses (PRISMA) chart detailing the workflow for the selection process of relevant studies. * The advanced search function of the Google Scholar database was used to collect references

The detailed workflow of the study selection process and the results obtained during the screening process are presented in Fig. 2. After formulating queries from all databases was *n* = 238 entries were obtained, with the highest number of studies gathered from Scopus (*n* = 88), PubMed (*n* = 83), Google Scholar (*n* = 41), and ScienceDirect (*n* = 26). After removing duplicate records from the databases, approximately (*n* = 171) were selected for title screening. Title screening was performed by examining each record in the database and assessing its potential relevance to the review topic. Studies that did not include terms related to HS or poultry were excluded, resulting in a refined database of (*n* = 112) studies. Further appraisal was conducted by reading the abstracts, which helped identify reviews, short communications, and other reports that did not include relevant bibliographical data for potential extraction, leaving (*n* = 66) studies for full scrutiny. Finally, each study was assessed based on predefined selection criteria to ensure qualification for inclusion in the systematic review. Finally, only (*n* = 18) studies were eligible for inclusion. Other studies were excluded because they considered HS mitigation strategies (*n* = 36), the experimental animals were not chickens (*n* = 7), and they did not include a control treatment in their design (*n* = 5).

### Description of the studies

#### Characteristics of experimental designs

Table 1 summarizes the characteristics of the experimental designs used in the selected studies. Although the 18 studies included in the review examined the effects of HS on the gut microbiome of chickens, they were conducted by diverse research groups with varying objectives. Consequently, HS protocols, experimental animals, and reported outcomes varied significantly. Regarding the HS protocol, the peak temperature recorded during the HS challenge across trials averaged approximately 33.6 °C, with a minimum of 30 °C and a maximum of 38.0 °C. All studies opted for prolonged exposure of the birds to high temperatures, with 14 choosing a cyclic HS approach and 4 consistently maintaining a chronic HS regimen. While studies that exposed birds to chronic HS maintained the temperature for 24 h daily, there were slight variations in the daily duration of HS exposure in cyclic HS experimental protocols. The average daily exposure time was approximately 8.42 h, with 11 studies choosing 8 h; the longest exposure time was 10 h and the shortest was 8 h. Furthermore, considerable diversity was observed among the experimental animals used. Fourteen studies used broilers, while only four used layers as experimental animals. Specifically, three studies that used layers chose the Hy-Line, whereas only one used the Isa-Brown. Conversely, a greater variety of broiler lines was used, with the most prevalent being yellow feathered (*n* = 6), followed by Arbor Acres (*n* = 5), multiple (*n* = 2), and Ross 308 (*n* = 1). As expected, the starting age of exposure was influenced by the category of birds used, ranging from several weeks for layers to a few days for broilers. The majority of studies, including fast-growing broilers, initiated HS exposure at 21 (*n* = 2) or 28 days (*n* = 6), whereas slow-growing broilers opted for approximately 56 days (*n* = 4). Only a few studies started exposure at 1 (*n* = 1) and 14 (*n* = 1) days of age. Regarding layers, none of the four studies used a similar starting age for HS exposure, which was 11, 12, 28, or 48 weeks.

#### Summary of the outcomes reported

A systematic overview of the parameters measured in each study is presented in Table 2. The investigations targeted three main sections of the gut: the cecum (*n* = 12), ileum (*n* = 3), and jejunum (*n* = 2). Only one study reported the presence of fecal material.

The objective of the current systematic review was to identify the associations between HS and changes in the gut microbiota; therefore, emphasis was placed on the parameters frequently reported in microbial ecology or composition analysis. Researchers have primarily assessed parameters such as alpha diversity, beta diversity, microbial composition, and microbial functional pathways. Microbial composition was the most frequently reported parameter (*n* = 18), followed by beta (*n* = 16) and alpha (*n* = 15) diversity, whereas only six studies (*n* = 6) analyzed microbial functional pathways (Table 2). After examining the texts of the studies, it was relatively straightforward to classify their results from alpha diversity as either showing an increase or decrease in indices related to richness and evenness, which was the first quantitative summary of the outcomes provided. Overall, most studies did not report significant changes in either the richness (*n* = 11) or evenness (*n* = 12) indices. In addition to the studies that did not assess any richness-related metrics, only an increase (*n* = 3) was reported. Similarly, two studies reported an increase in evenness indices, while only *n* = 1 reported a decrease; a comparable number of studies did not assess evenness indices. Results addressing the use of beta diversity were also compiled; however, due to the nature of the analysis (only reporting similarity between samples from different treatments), we only reported whether significant differences were found between the HS and control treatments with at least one beta diversity metric. It was found that a total of *n* = 15 studies reported significant differences induced by HS in beta diversity analysis. Only *n* = 2 studies did not report results, and one study did not find evidence of differences associated with beta diversity. Finally, it was not feasible to quantitatively assess the results related to the microbial composition and/or functional pathways. Many microbial taxa and functional pathways were affected by HS, and an overview of the main findings is shown in Table 3.

**Table 3:** Main findings reported by the authors in the studies included in the systematic review

Study	Main findings
Xing et al. [21]	No differences in alpha diversity; Significant differences in beta diversity; 15 genera identified as biomarkers of HS
Shi et al. [23]	Significant differences in beta diversity; Higher abundance of Firmicutes, Tenericutes, and Proteobacteria in HS samples; Lower Bacteroidetes, *Oscillospira*, and *Faecalibacterium* caused by HS
Wang et al. [24]	A total of 9 identified biomarkers for HS at the genus level; Significant differences in alpha and beta diversity; Overall increased richness associated with HS
Liu et al. [25]	Significant differences in alpha and beta diversities; 8 biomarkers of HS were identified at the genus level
Campos et al. [26]	HS induced interactive effects between line and richness; Significant differences in beta diversity; HS had led to interactive effects between microbial taxa abundance and line
Emami et al. [27]	Higher Shannon indices; Significant differences in beta diversity; HS affected microbial biomarkers of breeds differently
Jin et al. [28]	Similar alpha diversity; Reduced *Campylobacter* and increased *Delftia* abundance; HS affected functional pathways
Liu et al. [29]	*Anaerovorax* was identified as a microbial biomarker of HS; HS enriched 10 signaling pathways
Liu et al. [30]	HS reduced the number of microbial taxa at the class and order level; HS also reduced the abundance of *Ruminococcus*, *Bdellovibrio*, and *Serratia*
Liu et al. [31]	HS decreased the abundance of Firmicutes and increased Proteobacteria; HS also increased the abundance of harmful bacteria; KEGG enrichment of purine metabolism was associated with HS
Zhu et al. [32]	HS reduced Firmicutes and increased Bacteroidetes; HS led to the identification of genera associated with liver and intestinal dysfunction
Yuan et al. [33]	No impacts of HS on alpha and beta diversity; HS was associated with a higher abundance of *Enterococcus*, *Weissella*, and *Reyranella*, and lowered populations of *Romboutsia *and *Ruminiclostridium*
Seo et al. [34]	HS upregulated pathways related to lipid and protein metabolism; A total of 11 microbial taxa were identified as HS biomarkers including *Bacteroides* and *Peptostreptococcaceae*
Wang et al. [35]	HS led to decreased abundances of Firmicutes, and an increase in Proteobacteria populations
Wang et al. [36]	HS increased Peptococcaceae and decreased Christensenellaceae and Lachnospiraceae abundances; Genera *Ruminococcus* and *Clostridium* were identified as HS biomarkers
Yang et al. [37]	HS increased Firmicutes, *Escherichia/Shigella*, *Phascolarctobacterium*, *Parabacteroides*, and *Enterococcus* abundances; HS decreased *Barnesiella* and *Alistipes* populations
Zhang et al. [38]	Enrichment of *Peptoniphilus*, Firmicutes, and Clostridiaceae by HS
Zhou et al. [39]	HS increased Firmicutes to Bacteroidetes ratio; HS upregulated pathways involved in proteolysis; *Faecalibacterium* and *Methanobrevibacter* identified as HS biomarkers

## Discussion

### Brief reminder: general effect of heat stress on gut physiology

**Fig. 3:**
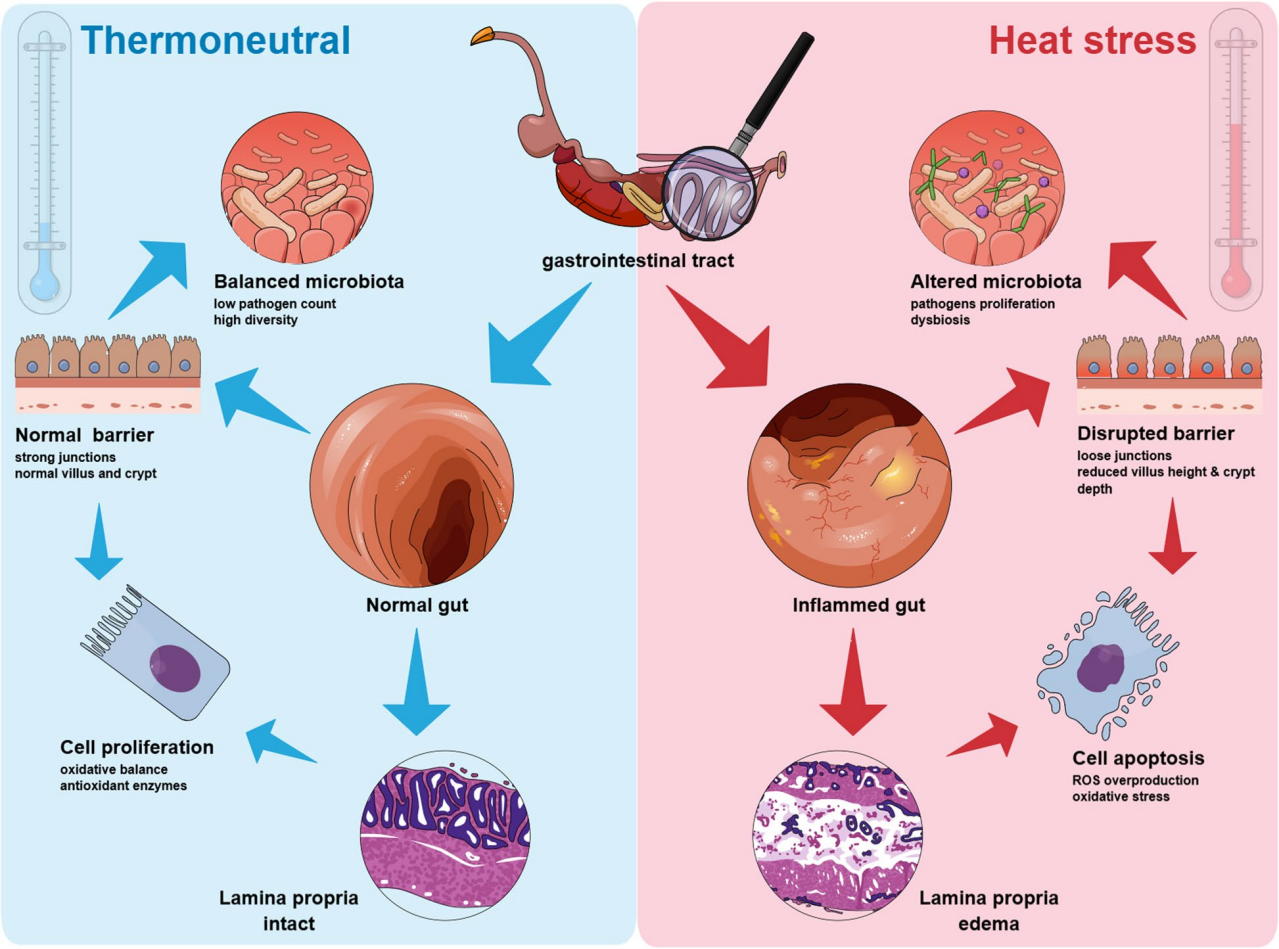
Selected and established effects of heat stress (HS) on the gut of chickens. HS affects intestinal integrity, leading to localized oxidative stress, pathogen colonization, and inflammatory reactions

The intestinal epithelium of chicken harbors diverse microbial populations. Thus, it is essential to thoroughly comprehend how HS affects the gut to accentuate its detrimental consequences on the intestinal microbiota. Notably, previous research has indicated that the influence of HS on the avian intestinal tract is complex, resulting in significant disruptions in gut integrity [40], immunology [41], and microbiota dynamics (Fig. 3).

The optimal functioning of a bird's gut is critical for maintaining homeostasis, as physical alterations to its structure can trigger a cascade of metabolic reactions that affect production performance and health [42]. Unfortunately, one of the first manifestations of HS in the poultry gut is alteration of intestinal morphology with increased intestinal permeability [43, 44]. Similar to other monogastric animals, the gut barrier of chickens is composed of tight junctions that ensure a firm connection between the enterocyte membrane and the protective mucosal layer [45, 46]. During HS, reduced feed intake, concomitant with hypoxia and ischemia, leads to destruction of the protective mucosal layer and loosening of tight junctions [10, 46]. This phenomenon, commonly known as leaky gut, is the primary cause of local inflammation reported in many studies [47]. Research has shown that intestinal permeability during HS is linked to the intra- and intercellular disruption of tight junctions. For instance, cyclic exposure to HS results in epithelial cell abscission, lamina propria edema, and inflammatory cell infiltration in the gut of broilers [25]. Other studies have also reported changes in intestinal morphometry, characterized by significantly decreased villus height and villus height-to-crypt depth ratio following cyclic HS in broilers [48]. These alterations were observed across all sections of the small intestine and did not appear to vary in magnitude, suggesting that the duodenum, jejunum, and ileum are equally vulnerable to HS.

HS has been reported to have a significant impact on gut health, particularly through localized oxidative stress [25]. Oxidative stress occurs when the overproduction of free radicals exceeds the capacity of the host antioxidant defense system to neutralize them [49]. This can lead to metabolic disruptions such as lipid peroxidation, DNA damage, and cell apoptosis [50]. In the gut, reactive oxygen species (ROS) and reactive nitrogen species (RNS) are produced either by intestinal epithelial cells or bacterial symbionts during their natural life cycle [51]. These ROS and RNS are usually scavenged by the organism's antioxidant defense enzymes, such as glutathione peroxidase (GSH-Px), superoxide dismutase (SOD), and catalase (CAT) [49–51]. However, during HS, hypoxia and energy deprivation in enterocytes exacerbate RNS and ROS production, and impair the activity of antioxidant enzymes, leading to oxidative injury [52]. The severity and duration of HS exposure appear to be primary factors governing oxidative injury. Both acute and chronic HS have been shown to reduce the expression of GSH-Px, SOD, and CAT and exacerbate lipid peroxidation in the gut of chickens [25]. For instance, exposure to HS for several consecutive weeks leads to higher levels of malondialdehyde (MDA) and suppresses SOD activity in the jejunal mucosa [53]. Similarly, acute HS for a few hours resulted in the downregulation of all antioxidant enzyme gene expression, accompanied by elevated levels of heat shock proteins (HSPs) in the ileum and duodenum of chickens [54]. Although both categories of HS can cause oxidative injury in the gut, there is a higher likelihood of recovery after acute HS because exposure usually does not last for more than 24 h. Birds often employ non-evaporative cooling mechanisms to cope with short periods of heat exposure [55]. By using simple postural adjustments, such as crouching or standing with their wings away from their body, they can mitigate heat buildup in the gut [9]. This behavioral adaptation increases the blood flow and heat transfer to the body surface, resulting in decreased heat loads within the internal organs [3]. In addition, following acute HS, there is typically an increase in feed intake within the next few hours, which enhances nutrient availability and promotes the gradual restoration of the gut structure over time.

### Synthesis of the presented evidence: impact of heat stress on the gut microbiota

In the existing literature, a limited number of studies have exclusively examined the impact of HS on the gut microbiota of chickens, without assessing the influence of potential mitigation strategies. Nevertheless, valuable insights into the modulatory effects of HS on the gut microbiota can still be obtained from these studies. Changes linked to microbiota dynamics during HS are also thought to be mediated by the microbiota-gut-brain axis. These mechanisms have been thoroughly detailed in a previous report [10] and are beyond the scope of this review. According to the studies included in this review, the effects of HS on gut microbiota have mostly been evaluated based on microbial diversity, composition, and functional pathways.

#### Heat stress and microbiota diversity

##### Alpha diversity

Alpha diversity has been extensively studied across various trials focusing on the impact of HS on chicken microbiota. Indeed, of the 18 studies included in the current review, only three did not report results related to alpha diversity. Commonly utilized indices, such as observed species, Chao1, Faith's phylogenetic diversity, Pielou's evenness, Shannon, and Simpson indices were employed to describe species richness and evenness [56]. As shown in Table 1, most of these studies have primarily focused on the small intestine, cecum, or, to a lesser extent, fecal samples. Although extensively studied, inconsistent findings have been reported on the effects of HS on species richness and evenness. Indeed, the majority of studies found a non-significant outcome while only three reported an increase in at least one of the metrics used to quantify species richness. Similarly, the results concerning evenness followed a comparable trend, as only two studies reported an increase and only one noted a decrease in the evenness indices. Several factors may have contributed to the observed disparity in outcomes, with the predominance toward no effect on alpha diversity. Indeed, other experimental variables with a more substantial impact on gut microbiota may have been the primary cause. For example, factors such as the initial state of the microbiota before heat exposure should not be disregarded. Evidence suggests that microbial communities exhibit varying levels of resilience, depending on their composition [57]. In experimental protocols for broilers, HS is typically applied from the third week of life, and the effects of a basal diet can influence the intestinal microbiota. These effects are more pronounced in laying hens, as trials usually start during the productive performance of birds and require several weeks to complete. Indeed, an examination of the average age at the onset of HS exposure across studies supports this hypothesis, as only one study applied HS from day one. Furthermore, it can be inferred that the overall duration and intensity of the HS challenges throughout the trials may have been insufficient to induce significant changes in alpha diversity. As shown in Table 1, 14 of 18 studies employed the cyclic HS approach, while only four consistently maintained birds under HS. Cyclic HS is a common protocol used in poultry studies and aims to simulate real-life temperature fluctuations by exposing birds to HS for a limited duration [58]. In this setup, exposure to HS is generally shorter than that under normal temperature conditions, potentially allowing birds sufficient time to cope with the extreme temperatures experienced during the day. Teyssier et al. [59] provided evidence that while cyclic and chronic HS impair the growth and meat quality of broilers, significantly worse effects were observed in birds subjected to chronic HS. This finding was corroborated by Souza et al. [58], who demonstrated that chronic HS leads to drastically reduced energy and nitrogen efficiencies, which are not perceptible under cyclic HS exposure. Although the general trend indicates non-significant results, it is important to acknowledge that a few studies have reported an increase in alpha diversity metrics, which should not be disregarded. Two of these studies simultaneously reported increased richness and evenness [27, 31]. One potential explanation for these observations may be the proliferation of pathogenic bacterial populations. Previous reviews support the hypothesis that pathogenic bacteria commonly found in avian species can be identified as microbial markers of HS, primarily because of an increase in their relative abundance [10]. Indeed, some studies have also revealed the presence of higher populations of *Escherichia coli*, *Salmonella *spp*.*, and lesser-known pathogenic bacteria, such as *Weissella *spp. [20, 33, 60] after exposure to HS. It has been hypothesized that HS impairs beneficial bacteria owing to limited nutrient availability while promoting pathogen proliferation because damage to the intestinal epithelium weakens its protective function [61]. Overall, although most of the studies included in this review indicated no strong evidence of the effect of HS on alpha diversity in the gut of birds, confounding factors may partially explain this tendency. However, contrasting results were observed when the beta diversity was considered.

##### Beta diversity

Beta diversity is the second most commonly used type of diversity in microbial community analyses. It is employed to differentiate microbial community structures between various treatments [62]. The results of the current review strongly support the notion that exposure to HS alters the microbial community structures in the gut. Indeed, 15 studies found evidence of significant beta diversity differences between the control and HS treatment groups. Notably, this difference in beta diversity was observed even in trials that did not demonstrate differences in alpha diversity or abundance of microbes at higher taxonomic ranks. For instance, beta diversity analyses revealed heat-induced microbial shifts in the cecum of broilers; however, no significant effect on the abundance of Bacteroidetes and Firmicutes was observed [21]. Similarly, Wang et al. [24] found a limited influence of HS on the relative abundance of major phyla in the ileum of broilers; however, significant clustering related to ambient temperature was observed. One potential explanation for this phenomenon is that HS affects the abundance of microbial taxa to varying degrees, with lower taxonomic ranks, such as species and genera, being more susceptible. It is well established that HS leads to an increased heat load within the internal organs of birds [63]. Although microbial species within a genus may share the same optimal growth temperature, this is less likely to be the case within a phylum. It is crucial to highlight that various metrics have been applied to differentiate between HS and thermoneutral-reared birds. Indeed, researchers have employed a range of metrics from the Bray–Curtis and Jaccard indices to more comprehensive metrics such as UniFrac distances [64]. UniFrac distances are phylogenetic measures that account for evolutionary relationships among microbial species [65]. Combined with these metrics, the choice of clustering technique significantly affected the observed microbial shifts in response to HS. For example, Liu et al. [31] used principal component analysis (PCA) with Euclidean distances to reveal distinct microbial community structures between HS and control treatments. Conversely, less pronounced distinctions were observed between clusters of HS and control birds in a study that employed UniFrac distances and principal coordinate analysis (PCoA) [26].

It is important to consider bird age as a potential explanatory factor. Shi et al. [23] demonstrated that the cecal microbiota of broilers exhibits stronger heat-induced shifts with increasing age. Indeed, HS had little effect on the microbial structure of 15-day-old broilers but resulted in completely distinct clusters in 42-day-old birds. It is well established that older birds are more susceptible to HS owing to their body weight and feather coverage, which affect their ability to dissipate heat [42]. Consequently, they cannot rely solely on behavioral changes to cope with HS. Additionally, the duration of exposure to HS may play a significant role in beta diversity assessment, highlighting the need for further research on the impact of short-term acute HS, as most studies included in this review involved cyclic HS applied for at least 14 d in broilers and several weeks in layers. Goel et al. [66, 67] investigated the effects of short-term acute HS on cecal microbial composition in broilers. Their findings did not reveal any contrast in beta diversity between thermoneutral birds and those subjected to HS. The results were consistent regardless of whether unweighted or weighted UniFrac distances were used for clustering. Conversely, 15 studies that reported significant differences in beta diversity considered either cyclic or chronic HS in their experimental designs. It can be inferred that the structure of the microbial communities underwent noticeable changes when prolonged periods of HS were applied. Overall, further longitudinal studies are necessary to fully examine the key factors influencing changes in the microbial structure of chickens exposed to high ambient temperatures.

#### Heat stress and microbiota composition

Microbiota composition was the most frequently analyzed parameter in the studies included in this review. Indeed, all studies provided at least some results related to the microbiota composition. To analyze the microbial composition among treatments, researchers typically employ non-parametric tests such as Kruskal-Wallis and Wilcoxon rank tests or more complex methods such as linear discriminant analysis effect sizes (LefSe) [68]. These techniques are designed to identify differences in the relative abundance of microbial taxa associated with a specific treatment, in this case, HS. Many authors have carried out these tests across different taxonomic ranks, from the phylum to the species level. Consequently, there is a vast array of reported outcomes, with some inconsistencies among the studies. Despite these discrepancies, the general trend indicates that HS is linked to changes in the relative abundance of specific microbial taxa, either increasing or decreasing regardless of the duration of exposure [69].

##### Changes at the phylum level

A common finding reported across studies that focused on changes occurring at the phylum level was that exposure to high ambient temperatures altered the relative abundances of Firmicutes, Bacteroidetes, Proteobacteria, and Tenericutes [23, 24, 31]. For instance, Liu et al. [31] noted a significant increase in the Proteobacteria population in the ceca of broilers subjected to HS for 14 days. This suggests that HS is associated with gut dysbiosis in chickens. Proteobacteria typically constitute a minor proportion of the balanced gut microflora in monogastric animals [70]. However, a previous report indicated that the expansion of facultative anaerobic bacterial populations in the host microbiome is a reliable indicator of gut dysbiosis [71]. It has been suggested that high Proteobacteria populations in the gut are indicative of intestinal ecological imbalance, which is commonly observed in pathogenic conditions such as colitis, inflammatory bowel disease, and diarrhea [72]. Other studies reported compromised growth and inflammatory reactions associated with an abnormally high abundance of Proteobacteria [73]. The increase in Proteobacterial abundance during HS may be an indirect consequence of oxidative stress. A previous study demonstrated a link between the proportion of Proteobacteria and the distribution of oxygen in the lower gut of humans [72]. Consequently, the accumulation of ROS such as superoxide anions may induce a shift in bacterial communities from obligate to facultative anaerobes, which constitute a substantial proportion of the Proteobacteria phylum [74]. A similar mechanism of action was also hypothesized to explain the phenomenon of “dysanaerobiosis” observed in patients with inflammatory bowel disease [75]. An increased relative abundance of Tenericutes was also found to characterize birds suffering from HS [23]. This change in microbial abundance may reflect the greater resilience of Tenericutes to environmental stress. The higher internal load and disrupted balance in the guts of birds exposed to HS may be one of the main factors associated with the decrease of commensal bacterial populations, such as *Lactobacillus* and *Bifidobacterium* observed previously [76]. Tenericutes are characterized by the presence of flexible cell membranes that provide resistance to environmental stressors. Therefore, it may be understood that as the overall microbial population in the gut declines following HS, Tenericutes can maintain their population, resulting in an overall greater relative abundance.

Unlike Proteobacteria and Tenericutes, the effect of HS on the relative abundances of Firmicutes and Bacteroidetes was not consistent. Some studies have indicated an increase in the Firmicutes population, whereas others highlighted a higher abundance of Bacteroidetes [23, 31]. However, it is important to note that changes in one phylum are often accompanied by opposing trends in another. Although not always significant, an increase in Firmicutes is frequently associated with a decrease in Bacteroidetes. Consequently, the Firmicutes/Bacteroidetes ratio may be a more practical parameter to assess [77]. Indeed, Zhou et al. [39] reported an increased Firmicutes to Bacteroidetes ratio in hens exposed to chronic HS. In addition, although not directly assessed, two studies reported a significant increase in the Firmicutes population with a concurrent reduction in Bacteroidetes in broilers, which could be interpreted as an elevated Firmicutes-to-Bacteroidetes ratio [23, 25]. Another study on the layers reported similar outcomes [21]. The Firmicutes/Bacteroidetes ratio has been established as a valid indicator of gut dysbiosis in humans and obese mice [78]. These findings have also been validated in patients with fatty liver diseases [79]. HS is a crucial factor that significantly affects the chicken carcass traits. Although the overall body weight of birds is drastically reduced under HS, long-term exposure to high ambient temperatures increases the abdominal, subcutaneous, and intramuscular fat deposition in chickens [80, 81]. The potential correlation between excess fat deposition and a higher Firmicutes to Bacteroidetes ratio can be primarily attributed to their different biological structures and functions. Firmicutes is a phylum of bacteria characterized by thick cell walls, primarily composed of peptidoglycans, and includes many Gram-positive species [82]. In contrast, Bacteroidetes possess thin cell walls and are predominantly Gram-negative [83]. The specificity of Firmicutes makes them more effective than Bacteroidetes in extracting energy from digesta, thereby promoting efficient absorption of calories and subsequent fat deposition [82]. However, it is important to note that the current literature remains controversial, as many studies have not directly measured the ratio, and sometimes contradictory results have been reported. For example, extended HS protocols in studies focusing on layers revealed significantly lower Firmicutes [31] or higher Bacteroidetes populations [32]. Nonetheless, these studies have consistently reported different microbial community structures between thermoneutral and HS birds. Thus, the focus should be on emphasizing that alterations in the initial population of these phyla are characteristic of HS. Overall, further studies should ideally be conducted, and a meta-analysis of microbiome data from chicken studies may provide a better overview of the relative abundance changes in both phyla owing to HS.

##### Changes at lower taxonomic levels

Although changes at the phylum level are often subtle, all studies included in this review identified at least a few significant changes in abundance at the genus or species level. For example, when evaluating the microbial composition changes between HS and thermoneutral birds, Shi et al. [23] reported a decreased population of the *Faecalibacterium* genus in the ceca of HS birds. The authors also highlighted a more pronounced reduction observed during prolonged HS exposure. In monogastric, *Faecalibacterium *spp*.* have been identified as key microbes in maintaining colonic mucosal health because of their ability to produce high levels of butyrate and crucial role in regulating tight junction gene expression [84]. *Faecalibacterium* has been consistently reported as one of the most dominant genera in chicken cecae [22, 85]. Additionally, previous studies have indicated that healthier broilers supplemented with vitamin additives exhibit a considerably higher proportion of *Faecalibacterium* [86]. It has also been suggested that certain *Faecalibacterium* species can be classified as beneficial bacteria in the gut of monogastric animals, and a decline in their populations is primarily observed during disease [87]. The detrimental impact of HS on the overall health of birds cannot be overstated (gut dysbiosis, inflammation) [42, 88]. This may explain the strong correlation between the reduction in *Faecalibacterium* and physiological alterations triggered by a wide range of diseases.

Another microbial taxon that was reportedly reduced drastically in the guts of chickens following exposure to high ambient temperature exposure was *Ruminococcus*. Liu et al. [30] reported a decrease in the relative abundance of *Ruminococcus* after HS in the jejunum of yellow broilers. It is already acknowledged that the gut microbiota is essential for combating pathogens and maintaining intestinal integrity [10]. The jejunal microbiota is particularly sensitive, as it can crosstalk with the barrier integrity and immune functions [3]. Evidence suggests that *Ruminococcus* plays a role in protecting the jejunal mucosa. Indeed, by metabolising cellulose and other polysaccharides, *Ruminococcus* species produce beneficial volatile fatty acids, such as butyrate, acetate, and propionate [89]. Additionally, they are strictly anaerobic, Gram-positive, non-motile cocci that require fermentable carbohydrates for growth [89]. Therefore, lower nutrient absorption combined with oxidative stress commonly observed in the intestines of birds exposed to HS may explain the reduction of *Ruminococcus* observed. Another study reported a decreased abundance of *Ruminococcus* in the ruminal fluid of beef cattle exposed to HS. Similarly, the authors justified their findings by attributing lower feed intake to HS exposure, which resulted in low fiber content in the rumen [90].

Microorganisms from the genus *Campylobacter* are well known for their capacity to colonize the intestinal tract of chickens at an early stage, and their populations tend to persist throughout their lifespan [91, 92]. Although these microorganisms are harmful to humans, they are considered commensals in avian species owing to their highly adaptable nature to their host [91]. According to Jin et al. [28], chronic HS exposure led to a notable reduction in the population of *Campylobacter* in the ileum of yellow broilers. The authors argued that their findings may be directly related to the prediction of the metabolic function of the intestinal microflora. In their study, HS increased carbohydrate metabolism, whereas the opposite was observed for amino acid metabolism. Because *Campylobacter* prefers to use amino acids such as aspartic acid, serine, and glutamic acid [93], the observed reduction in their population during HS might be associated with lower amino acid metabolism. Another possible reason for the reduction in the *Campylobacter* population could be destruction of the intestinal mucosal layer caused by HS. It is believed that the adaptation of *Campylobacter* to chicken hosts is facilitated by strong adhesion to epithelial cells [94]. There is evidence that *Campylobacter* binds to fibronectin, a particularly abundant glycoprotein in the extracellular matrix of the gut of chickens [91]. Therefore, epithelial cell apoptosis induced by HS may lower the adherence of *Campylobacter* spp. to the gut of birds.

It is also assumed that HS influences the intestinal microbiota of chickens by promoting the growth of pathogens [10]. Pathogenic bacteria commonly found in avian species have been identified as microbial markers of HS primarily because of an increase in their relative abundance [61]. *Escherichia coli* is one of the most frequently reported pathogens that exhibits strong interactions with HS. In chickens, *E coli* is a pathogenic intestinal bacterium that causes colibacillosis, which results in severe morbidity and mortality in flocks [95]. Studies have shown that HS exacerbates *E. coli-*induced intestinal inflammation in chickens. Tang et al. [96] reported that HS amplified the effects of *E. coli* on intestinal inflammation in pure-line Ma chickens. Similarly, Zhang et al. [97] highlighted that HS led to an increase in the population of *E. coli* within the intestinal microflora of broilers. Additionally, Park et al. [98] found that extreme HS conditions resulted in a higher abundance of *Escherichia *spp. in the ceca of Ross broilers. Other species of the *Enterobacter* genus have also been shown to exhibit increased abundance during HS. Similarly, two recent studies reported a higher population of *Shigella *spp*.* in heat-stressed Ross broilers [37, 99]. Furthermore, a few studies that did not use metataxonomic analysis reported a link between *Salmonella* spp. intestinal colonization and HS [20, 60]. Additionally, lesser-known pathogenic bacterial populations were amplified under HS conditions. One such case was illustrated in a report that presented evidence of an increased abundance of *Weissella* spp. in the cecal microbiota of broilers exposed to heat stress [33]. Although the entire genus contains species with different relationships to their hosts, some strains of *Weissella* have been reported to be opportunistic pathogenic bacteria [100]. In conclusion, the intestinal epithelium, which serves as the first line of defense against pathogenic microbes, may no longer be able to fully perform its role as HS, results in severe damage to enterocytes and tight junctions. Additionally, the growth of beneficial bacteria may be impaired owing to limited nutrient availability, which can exacerbate the invasion of pathogens and lead to systemic infections, which are often observed in chicken flocks reared under high ambient temperatures.

#### Heat stress and microbial functional pathways

Examination of the studies included in this review revealed that only one-third (*n* = 6) reported data related to functional pathway analysis. Nevertheless, it can serve as a valuable tool for exploring the association between HS physiology and the gut microbiota. The method involves using gene prediction tools that have been used to assemble sequences and annotate them using databases such as the Kyoto Encyclopedia of Genes and Genomes (KEGG) [101], Clusters of Orthologous Groups (COG) [102], and MetaCyc [103]. The functional profiles of the HS and control groups were compared, which led to the identification of differentially abundant pathways.

The data obtained from synthesizing the current evidence indicate that metabolites involved in the tricarboxylic acid (TCA) cycle are the most affected by exposure to HS. One example is the decrease in the pathways related to the TCA cycle observed by Campos et al. [26] in two chicken lines. In addition, Liu et al. [29] provided several other specific examples using metabolomic and functional pathway analyses. They found that L-malic acid and citric acid, both of which are important in the TCA cycle, were reduced in the cecum of broilers exposed to HS. L-Malic acid has been reported to possess antimicrobial properties and beneficial effects on gut health in quails [104]. Similarly, citric acid has been found to positively affect the gut health of broilers when used as a dietary supplement [105]. Based on these findings, it can be inferred that HS harms chicken gut health, as evidenced by its effect on the TCA cycle through specific metabolites. The primary function of the TCA cycle is to oxidize acetyl-CoA to produce reducing equivalents that fuel the electron transport chain for ATP production [106]. As a result, reduced energy intake and hypoxia in the guts of birds exposed to HS can lead to ATP depletion, as shown by microbial functional pathway analysis.

As anticipated from the included studies, various pathways reportedly influenced by HS were associated with intestinal antioxidant functions and DNA damage. However, there appears to be a distinct difference in the regulation of these pathways, as those associated with the production of antioxidant metabolites are generally upregulated. One of the initial responses of an organism to HS is to maintain homeostasis by releasing endogenous antioxidants to neutralize excess free radicals generated by accumulated heat loads [107]. Such instances were observed in a study by Campos et al. [26] who noted a significant increase in the abundance of multiple pathways involved in ubiquinol synthesis. Ubiquinol is a lipid-soluble antioxidant that can be produced de novo in animal cells to prevent lipid peroxidation in biological membranes [108]. It is well established that HS leads to the overproduction of ROS, which can cause lipid peroxidation and damage cell membranes [3, 42]. Thus, the increased abundance of several pathways involved in ubiquinol synthesis may be a response to manage excess free radicals in cells [109]. Similarly, a study conducted by Liu et al. [31] revealed that KEGG enrichment results showed a greater abundance of differential metabolites involved in purine metabolism and ATP-binding cassette (ABC) transporters. This observation may be related to the findings of Campos et al. [26] and can be interpreted as a response to heat-induced oxidative stress. Purine is an essential component of DNA and RNA and is required for cell proliferation and survival [110, 111]. Another notable example from a study by Yang et al. [37] reported that pathways linked to ABC transporters are differentially enriched following HS. As previously highlighted by Campos et al. [26], these findings can also be explained by excessive ROS production linked to ABC transporter deficiencies. These molecules are crucial for maintaining oxidative balance due to their role in efflux of toxic compounds and transport of antioxidants, such as glutathione, across cell barriers [112]. Therefore, the observed enriched relative abundance was likely a concomitant response to oxidative stress.

Other studies have also emphasized the regulation of amino acid metabolic pathways by HS. However, it is important to note that the outcomes are not consistent across studies, which may be attributed to variables such as the type of chicken (hens, broilers, and dual usage) and lines (fast or slow growing). As demonstrated by Zhu et al. [32], the KEGG functional prediction suggested that the cysteine and methionine pathways were more prevalent in laying hens subjected to HS. This finding correlates with the increased methionine requirement in layers in hot climates or during extended summers [113]. Previous studies have confirmed these hypotheses by arguing that increased dietary methionine during HS is advantageous because it is one of the major essential amino acids that promotes egg production [114]. However, a counterexample was reported by Campos et al. [26], who found a decreased relative abundance of pathways related to methionine and tryptophan in different broiler lines. This contradicts the findings of Zhu et al. [32], although many authors have reported that HS alleviates the effects of methionine supplementation in broilers. Seo et al. [34] reported that tryptophan metabolism was among the upregulated pathways in the intestines of Ross 308 broilers exposed to HS. Therefore, the current literature does not provide clear conclusions regarding the amino acid metabolic pathways upregulated by HS.

In addition to the KEGG pathways commonly reported to be influenced by HS across various studies, a few others have been mentioned on a single occasion. Although mentioned only once, plausible explanations exist for the mechanisms underlying these findings. For instance, an increased abundance of pathways related to heme biosynthesis has been observed in jungle fowl reared under HS [26]. Heme is an essential cofactor for oxygen transport and activation in animal tissues [115]. Prolonged HS exposure leads to hypoxia in the intestinal mucosa, subsequently resulting in a leaky gut [3, 10]. Therefore, one of the initial behavioral responses to HS in birds is panting, which serves a cooling function and provides sufficient oxygen to tissues that may be oxygen-deprived [116]. Seo et al. [34] provided further examples of the specific pathways modulated by HS. Their study revealed that the fatty acid degradation-related pathways were significantly upregulated on HS exposure. Enhanced fatty acid degradation in the jejunum of birds is associated with digestion and may play a role in the adaptive physiology of birds by increasing energy metabolism under high-temperature loads [117]. These observations may also be linked to the upregulation of pathways related to ROS production as observed in earlier studies, as lipid degradation promotes peroxide formation, which causes apoptosis due to DNA damage [118]. As research in this area continues, more evidence will likely become available and the mechanisms underlying the modulation of specific pathways will be further elucidated.

### Quality of the presented evidence, limitations, and future directions

According to the literature, the effect of HS on the gut microbiota of chickens remains an area of active research and development. One of the biggest challenges facing researchers in this field is the limited availability of information on specific parameters such as microbial functional pathways. Additionally, while there has been an effort to report findings, there is still an overemphasis on certain organs, with the cecum being the most studied because of its functions and higher microbial populations. Another issue is the poor description of sequencing techniques and data processing pipelines, resulting in inconsistent reporting even from studies conducted by the same research groups. In fact, research has also demonstrated that microbiota analysis results, such as those discussed in the current review, can exhibit significant variation based on the DNA isolation methodology, sequencing region selection, and data analysis approach. Furthermore, research designs often rely on cyclic HS with slight variations, which may limit our current understanding of the associations between host core body temperature variations and the gut microbiota. The low reproducibility of experimental trials is also a significant limitation, as factors such as diet and environmental conditions can greatly affect the intestinal microbiota signature of hosts. Finally, although this review attempted to offer a comprehensive synthesis of literature, it included only 18 studies. This was primarily attributable to the strict inclusion criteria. Specifically, only studies that exclusively evaluated the impact of HS were selected, because researchers often focus on parameters known to be influenced by their mitigation strategies. For example, trials involving supplements with known antioxidative properties are likely to include parameters associated with antioxidant pathways or to correlate microbial taxa abundance with oxidative stress markers. However, it is important to recognize that incorporating a greater number of studies might have provided additional insights into the mechanism of action of HS on chicken microbiota.

Future studies should consider clear directions for the current state of research. First, longitudinal and cross-sectional studies are needed to elucidate the impact of early and late HS exposure on microbial community dynamics across various segments of the gut. These study designs have been previously applied to investigate the core microbiota of chickens and have yielded insightful results. Chicken microbiota exhibits more stochastic than deterministic evolution throughout the lifetime of the host [119]. A similar methodology was recently employed to comprehensively describe the effects of age and intestinal sample sites on the impact of feed additives on the microbiota. It was discovered that the effects of antibiotic growth promotants (AGPs) do not induce identical microbial shifts in the proximal and distal parts of the GIT of birds. Additionally, host age appears to be a more significant factor influencing microbiota composition than AGPs [120]. Therefore, gaining a comprehensive understanding of microbial dynamics in the GIT when birds are subjected to HS would be an invaluable asset in devising probiotic-based mitigation strategies. These interventions may lead to increased effectiveness because they are tailored to the current state of the microbiota. Secondly, various HS protocols are commonly used in poultry trials, which may not result in similar microbial shifts. Acute HS refers to a brief period of intense heat exposure, whereas cyclic HS is often utilized to mimic diurnal temperature variations in hot climates. Chronic HS represents the most extreme form of stress, as birds are subjected to continuous high temperatures for extended periods. Studies have shown that birds do not react uniformly to all HS protocols and that microbial populations are likely to exhibit dissimilar responses. Acute HS causes less damage to the intestinal epithelium than that caused by chronic or cyclic HS [61]. Cyclic HS also permits daily recovery periods, resulting in synchronized variations in antioxidant enzymes and stress hormones [121]. Therefore, further research is required to improve our understanding of these phenomena. Additionally, there is a lack of studies on laying hens, and more research is needed to unravel the differences in microbiota composition between broilers and hens after exposure to HS. As previously mentioned, the changes observed in the microbiota of birds following HS appeared to be influenced by their initial microbiota before exposure. It is crucial to differentiate these production systems to develop suitable mitigation strategies, and it is essential to conduct trials with different lines to identify the potential differences among them. Finally, meta-analyses of microbiome data could help generalize our knowledge of the current literature, as several disease-specific and shared microbial shifts have been identified in humans using this approach [122]. Although common meta-analysis approaches are gaining popularity in animal science [123, 124], microbiome-based meta-analyses remain scarce.

## Conclusions

Although the available literature on the modulation of chicken intestinal microbiota by HS is limited, important findings can be obtained. Physical alterations associated with HS also lead to concomitant changes in gut microbial populations. Diversity indices and associated visualization techniques revealed shifts in microbial community structure following prolonged exposure to HS. Although inconsistent across studies, the microbial composition was generally altered, with changes at lower taxonomic ranks being more prevalent. There is also evidence for the modulation of microbial functional pathways associated with nutrient absorption and antioxidant biosynthesis. However, the mechanisms governing HS-induced microbial shifts remain poorly understood, and most studies have focused on the cecum as the investigation site. Overall, further research is required to improve our current understanding of the interaction between HS and poultry gut microbiota.

## Data Availability

Not applicable.

## Abbreviations

AGPs: Antibiotic growth promotants
CAT: Catalase
COG: Clusters of Orthologous Groups
GIT: Gastrointestinal tract
GSH-Px: Glutathione peroxidase
HS: Heat stress
HSPs: Heat-shock proteins
KEGG: Kyoto Encyclopedia of Genes and Genomes
LefSe: Linear discriminant analysis effect sizes
MDA: Malondialdehydes
PCA: Principal component analysis
RNS: Reactive nitrogen species
ROS: Reactive oxygen species
SOD: Superoxide dismutase
TCA: Tricarboxylic acid
VFA: Volatile fatty acid
